# Supplementary material for: Causal Relationship between Aging and Anorexia Nervosa: A White-Matter-Microstructure-Mediated Mendelian Randomization Analysis
Source: Biomedicines. 2024 Aug 16;12(8):1874. doi: 10.3390/biomedicines12081874 (PMC11351342; doi:10.3390/biomedicines12081874)
Supplement: Supplementary file 1 [file biomedicines-12-01874-s001.zip › Supplementary Figure/Figure S4/Singe SNP plot of MR analysis for TL to ubm-b-1723.pdf]

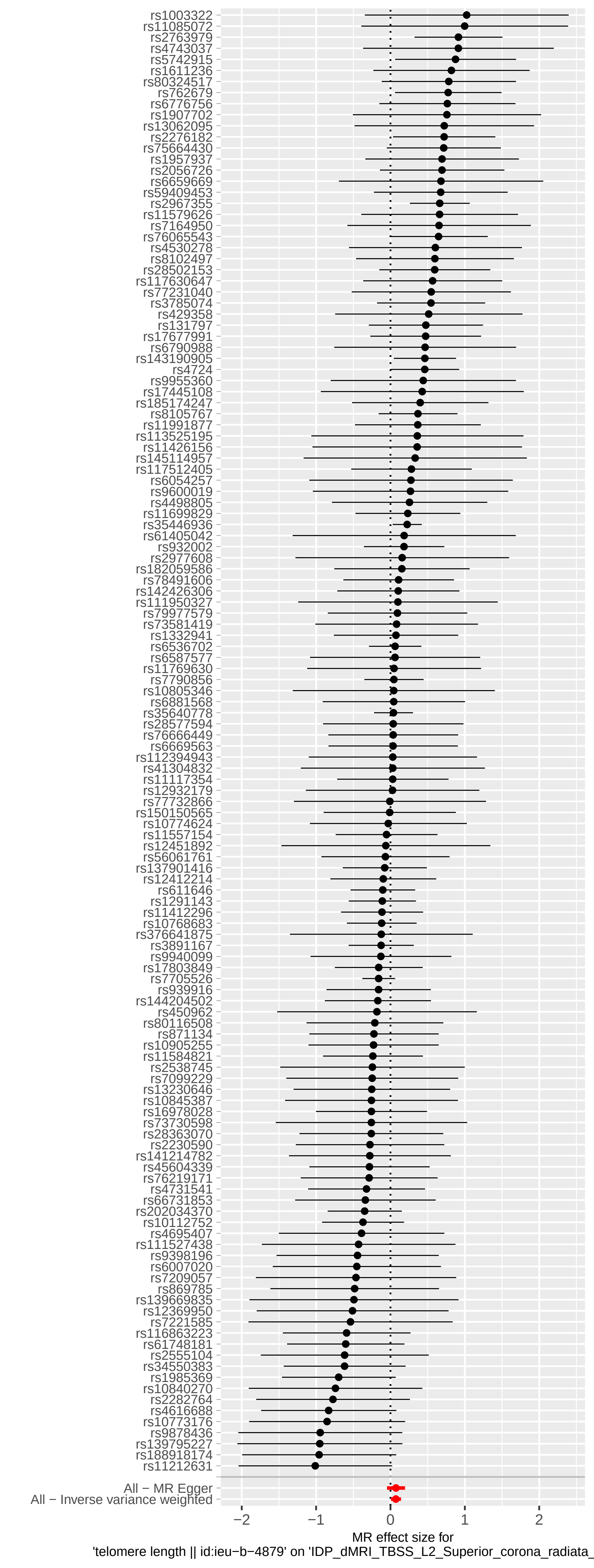

rs1003322
rs11085072
rs2763979
rs4743037
rs5742915
rs1611236
rs80324517
rs762679
rs6776756
rs1907702
rs13062095
rs2276182
rs75664430
rs1957937
rs2056726
rs6659669
rs59409453
rs2967355
rs11579626
rs7164950
rs76065543
rs4530278
rs8102497
rs28502153
rs117630647
rs77231040
rs3785074
rs429358
rs131797
rs17677991
rs6790988
rs143190905
rs4724
rs9955360
rs17445108
rs185174247
rs8105767
rs11991877
rs113525195
rs11426156
rs145114957
rs117512405
rs6054257
rs9600019
rs4498805
rs11699829
rs35446936
rs61405042
rs932002
rs2977608
rs182059586
rs78491606
rs142426306
rs111950327
rs79977579
rs73581419
rs1332941
rs6536702
rs6587577
rs11769630
rs7790856
rs10805346
rs6881568
rs35640778
rs28577594
rs76666449
rs6669563
rs112394943
rs41304832
rs11117354
rs12932179
rs77732866
rs150150565
rs10774624
rs11557154
rs12451892
rs56061761
rs137901416
rs12412214
rs611646
rs1291143
rs11412296
rs10768683
rs376641875
rs3891167
rs9940099
rs17803849
rs7705526
rs939916
rs144204502
rs450962
rs80116508
rs871134
rs10905255
rs11584821
rs2538745
rs7099229
rs13230646
rs10845387
rs16978028
rs73730598
rs28363070
rs2230590
rs141214782
rs45604339
rs76219171
rs4731541
rs66731853
rs202034370
rs10112752
rs4695407
rs111527438
rs9398196
rs6007020
rs7209057
rs869785
rs139669835
rs12369950
rs7221585
rs116863223
rs61748181
rs2555104
rs34550383
rs1985369
rs10840270
rs2282764
rs4616688
rs10773176
rs9878436
rs139795227
rs188918174
rs11212631
All − MR Egger
All − Inverse variance weighted
−2
−1
0
1
2
MR effect size for
'telomere length || id:ieu−b−4879' on 'IDP_dMRI_TBSS_L2_Superior_corona_radiata_
